# “I Can’t See an End in Sight.” How the COVID-19 Pandemic May Influence Suicide Risk: A Qualitative Study

**DOI:** 10.1027/0227-5910/a000877

**Published:** 2022-08-19

**Authors:** I-Ting Hwang, Fortune Fu-Tsung Shaw, Wen-Yau Hsu, Guang-Yi Liu, Chen-I Kuan, David Gunnell, Shu-Sen Chang

**Affiliations:** ^1^ Institute of Health Behaviors and Community Sciences, College of Public Health, National Taiwan University, Taipei, Taiwan; ^2^ Department of Counseling Psychology and Human Resource Development, National Chi Nan University, Nantou, Taiwan; ^3^ Department of Psychology, National Chengchi University, Taipei, Taiwan; ^4^ Research Center for Mind, Brain and Learning, National Chengchi University, Taipei, Taiwan; ^5^ Institute of Health Policy and Management, College of Public Health, National Taiwan University, Taipei, Taiwan; ^6^ Centre for Academic Mental Health, Population Health Sciences, University of Bristol, UK; ^7^ National Institute of Health and Care Research Biomedical Research Centre at the University Hospitals Bristol and Weston NHS Foundation Trust and the University of Bristol, UK; ^8^ Global Health Program, College of Public Health, National Taiwan University, Taipei, Taiwan; ^9^ Psychiatric Research Center, Wan Fang Hospital, Taipei Medical University, Taipei, Taiwan

**Keywords:** COVID-19, suicide, psychological responses, mental health, helpline

## Abstract

**Abstract:** *Background:* The COVID-19 pandemic and its consequences may affect population mental health and suicide risk. *Aims:* To explore the experiences among suicidal individuals who made calls to a suicide prevention hotline and to identify factors and psychological responses that may influence suicide risk. *Method:* We identified 60 eligible recorded calls to Taiwan’s suicide prevention hotline (January 23, 2020–May 31, 2020) and analyzed the transcripts using a framework analysis. *Results:* We identified three themes: (a) effects of the COVID-19 pandemic on society (impacts on local economies, the fear of contagion, and disruptions caused by outbreak control measures); (b) stress experienced by callers, including increased challenges (financial burden, restricted freedom of movement, interpersonal conflicts, feelings of uncertainty, and education/career interruption) and reduced support (reduced access to health services and social support); and (c) the callers’ psychological responses to stress, including anxiety, sleep disturbance, depression, loneliness, hopelessness, and entrapment, which may increase suicide risk. *Limitations:* Only the experiences among those who sought help by calling the hotline during the early months of the pandemic in 2020 were explored. *Conclusion:* Our findings revealed the potential process underlying the impact of the COVID-19 pandemic on suicide risk and have implications for prevention and intervention strategies.

The COVID-19 pandemic, government-imposed control measures, and their social and economic impacts may adversely affect population mental health (Arendt et al., 2020; Ettman et al., 2020; Pierce et al., 2020). Therefore, there are concerns that the suicide rate may increase in response to the pandemic and its consequences and calls for more research to examine whether and how the pandemic may influence suicide risk (Devitt, 2020; Gunnell et al., 2020; Holmes et al., 2020; Niederkrotenthaler et al., 2020; Reger et al., 2020).

Research to date has shown various findings of trends in different suicidal behaviors during the pandemic (John et al., 2020). Some longitudinal studies showed that the prevalence of suicide ideation and psychological distress increased during the pandemic (Czeisler et al., 2020; Niedzwiedz et al., 2021; O'Connor et al., 2020). By contrast, based on data from 21 mostly high-income countries, Pirkis et al. (2021) found that suicide rates remained unchanged or slightly decreased in the early months of the pandemic. This lack of effect on suicide rates may, in part, reflect the well-recognized influence on suicide rates of increased social cohesion (Durkheim, 1897/1951). However, more recent data from Japan showed that suicide rates increased in the second half of 2020, following an initial decrease during the COVID-19 pandemic (Tanaka & Okamoto, 2021). Since the effects of the pandemic on mental health and suicide risk could be complex and unfold over time, there is a need to deepen our understanding regarding how the pandemic might influence individuals’ suicide risk (McIntyre et al., 2021; Tanaka & Okamoto, 2021).

Researchers have suggested ways that the pandemic and its consequences may influence the pattern and frequency of suicidal behavior. Gunnell et al. (2020) and Niederkrotenthaler et al. (2020) pointed out a range of COVID-19-related factors that may increase suicidal behaviors, such as financial stress, domestic violence, and reduced social participation, as well as high-risk groups that may be particularly affected, such as individuals with pre-existing mental illness and those with a history of suicidal behavior. Empirical research so far has provided some information about how the pandemic may influence suicide risk. A recent systematic review summarized findings from 78 studies of trends in suicidal thoughts and behavior and factors associated with increased suicide risk during the COVID-19 pandemic (John et al., 2020). These studies revealed a range of factors such as fear of contagion, financial issues, quarantine, stress among health professionals, uncertainty in the progression of the pandemic, disruption to the regular routine, reduced social connection in some groups such as young people and migrants, arguments with family members, and feelings of isolation, loneliness, and entrapment. Most of these studies used data extracted from news reports of suicide deaths or data collected by clinicians who assessed individuals with suicidal ideation or behavior. However, these studies provided limited information about how the potential risk factors identified may interact and contribute to increased suicide risk in the context of the pandemic. No study has explored how pandemic-related stress and individuals’ psychological responses would contribute to the emergence of suicidal ideation based on the perspectives of those affected.

Psychological models of suicidal behavior, such as the interpersonal theory of suicide (Joiner Jr. & Rudd, 1996) and the integrated motivational‒volitional model of suicidal behavior (O'Connor & Kirtley, 2018), have provided valuable insights into how stress and associated psychological responses may contribute to suicidal ideation and behavior (O'Connor & Nock, 2014) and could inform the understanding of the potential impacts of the COVID-19 pandemic on individuals’ stress and suicide risk. However, we need to be aware that these models were developed in certain (mostly Western) contexts and may not be directly applicable in other cultural settings. Previous studies showed that the pattern of suicidal behavior in non-Western countries might differ from that in Western countries (Canetto, 2015; Chen et al., 2011; Coskun et al., 2012; Snowdon, 2018) and that the frequency, precipitating factors, methods, meanings, and consequences of suicidal behavior vary across cultures (Canetto, 2021). In addition, the impacts of the COVID-19 pandemic on mental health and suicide risk may also vary across societies and countries. Therefore, there is a need to conduct studies to obtain an in-depth and nuanced understanding of individuals’ lived experiences and psychological responses in relation to suicide risk during the pandemic in different cultural settings.

The first COVID-19 case was identified in Taiwan (population: 23 million) on January 21, 2020. Taiwan is close to the epicenter of the COVID-19 outbreak in China, and Taiwan’s government responded rapidly to the pandemic. With a combination of case-based (including contact tracing and quarantine) and population-based (including border control, physical distancing, and face masks) interventions (Ng et al., 2021), Taiwan was initially successful in containing the COVID-19 outbreak with a total of only 799 cases and seven deaths in 2020. However, government-imposed outbreak control measures still led to disruptions to people’s daily lives. Furthermore, as was the case in other countries, Taiwanese people also experienced a high level of uncertainty and anxiety during the early months of the outbreak (Wong et al., 2020). The perceived fear of people in Taiwan may have also been heightened by the experience of SARS in 2003, which resulted in 73 deaths among 347 confirmed cases (a high fatality ratio of 21%; Taiwan Centers for Disease Control, 2021). Nevertheless, recent studies from Taiwan showed no increase in overall suicide rates during the SARS epidemic (Chang et al., 2022) and a small reduction in overall suicide rates during the first year of the COVID-19 pandemic (Lin et al., 2021).

Taiwan’s national suicide prevention hotline, founded in 2009, is an important source of emotional support for people in times of crisis. The hotline service is provided by the Taipei Lifeline Association and funded by Taiwan’s Ministry of Health and Welfare. The hotline offers a 24-h toll-free number service for counseling and crisis intervention. The hotline helpers are trained to use active listening and the problem-solving approach when supporting callers. In 2020, the hotline received 104,501 calls, which is a 14% increase compared to that in 2019 (Taipei Lifeline Association, 2021).

This study aimed to explore the experiences among suicidal individuals affected by the COVID-19 pandemic and made calls to Taiwan’s national suicide prevention hotline during the early months of the pandemic and to identify factors and psychological responses that may influence suicide risk.

## Methods

### Study Design

We conducted a qualitative analysis of existing recorded conversations of COVID-19-related calls by suicidal individuals to Taiwan’s national suicide prevention hotline between January 23 and May 31, 2020. Qualitative approaches allowed us to obtain an in-depth understanding of lived experiences during the pandemic from the perspectives of affected individuals and explore important factors influencing individuals’ likelihood of engaging in suicidal behaviors (Hjelmeland & Knizek, 2010).

Certain characteristics of the calls, such as date, time, and call duration, were automatically captured by a computerized information system. Other characteristics, such as the callers’ gender, age, main concerns, previous suicide attempts, recent suicidal ideation, and immediate suicide risk (i.e., any current suicidal plan or action), were recorded by the hotline helpers.

Ethical approval for this study was granted by The Research Ethics Committee C, National Taiwan University Hospital (Reference Number 202004065RINC). As part of the hotline practice, the helpers asked for and obtained the callers’ consent for recording. The ethics committee deemed our study as secondary data analysis and agreed that our data management and analysis could ensure the protection of anonymity and confidentiality.

From January 23, 2020, shortly after the first COVID-19 case was identified in Taiwan on January 21, 2020, the Ministry of Health and Welfare requested the hotline helpers to mark calls in which COVID-19 was mentioned to monitor the potential mental health impact of the outbreak. During the study period, 3,471 calls were marked as COVID-19-related. We applied the following inclusion criteria to select calls for data analysis: (1) the callers reported suicidal behaviors or preparations for suicide while calling or the callers expressed that they always (i.e., every day) or usually (i.e., every week) had suicidal ideation and (2) the callers’ main concerns were related to the COVID-19 pandemic. Among the 3,471 COVID-19-related calls, 136 calls met the inclusion criterion (Criterion 1) according to the helpers’ notes. Among these calls, the first author, who has a clinical background in occupational therapy and experiences in conducting qualitative studies, listened to the audio recordings and determined if the callers’ main concerns were related to the COVID-19 pandemic (Criterion 2). In total, 60 calls met both the inclusion criteria. The characteristics of these selected calls are shown in Table 1.

**Table 1:** Characteristics of selected calls: caller demographics, mental health, history of previous use of the helpline, and call duration

Caller’s characteristics	Selected calls	
	(*n* = 60)	
	*n*	(%)
Sex		
Male	20	(33.3)
Female	40	(66.7)
Age group (years)		
<25	2	(3.3)
25–34	14	(23.3)
35–44	10	(16.7)
45–54	18	(30.0)
≥55	13	(21.7)
Unknown	3	(5.0)
Suicidal ideation		
Always (daily)	24	(40.0)
Usually (weekly)	34	(56.7)
Sometimes (biweekly)	1^a^	(1.7)
Occasionally (monthly)	1^a^	(1.7)
None or unknown	0	(0.0)
Previous suicide attempt		
Yes	40	(66.7)
No or unknown	20	(33.3)
Pre-existing mental health condition		
Yes	44	(73.3)
No or unknown	16	(26.7)
Call history		
First-time caller	26	(43.3)
Not the first-time caller or unknown	34	(56.7)
Call duration (minutes)		
2–10	7	(11.7)
11–20	20	(33.3)
21–30	17	(28.3)
≥31	16	(26.7)
*Note.* ^a^The two callers were included because they were having suicidal behaviors or preparing to have suicidal behaviors.		

### Data Analysis

To explore the callers’ experiences during the early months of the pandemic and to identify factors and psychological responses that may influence suicide risk, we conducted a framework analysis, which included seven stages (Gale et al., 2013).

After the 60 selected calls were deidentified and transcribed verbatim (Stage 1), the first author familiarized herself with the transcripts by reading and re-reading each transcript and taking notes (Stage 2). In the process of coding (Stage 3) and developing a working analytical framework (stage 4), the first author applied both deductive and inductive approaches. In the deductive phase, the first author listed an initial set of codes, which were broadly informed by the psychological theories of suicidal behavior (Joiner Jr. & Rudd, 1996; O'Connor & Kirtley, 2018; O'Connor & Nock, 2014) and potential risk factors for suicide during the COVID-19 pandemic suggested by researchers (Gunnell et al., 2020; Niederkrotenthaler et al., 2020). In the inductive phase, the first author did line-by-line coding and attempted to identify new codes that were not captured in the deductive phase. To enhance the credibility of the analysis, the last author, a board-certified psychiatrist and an expert consultant to the national suicide prevention hotline, reviewed the process of developing the analytical framework. The second author, a psychologist and an expert consultant to the national suicide prevention hotline, has first-hand experiences answering the calls as a volunteer hotline helper; he provided feedback to refine the codes and categories. After discussion, the analytical framework was finalized. The first author then applied the analytical framework to each transcript (Stage 5) and *charted* the data into the framework matrix (Stage 6) using the qualitative analysis software package ATLAS.ti 8. The research team members met as a group to identify themes, examine the relationships between themes, and explore how different themes were related to suicide risk (Stage 7).

## Results

**Figure 1:**
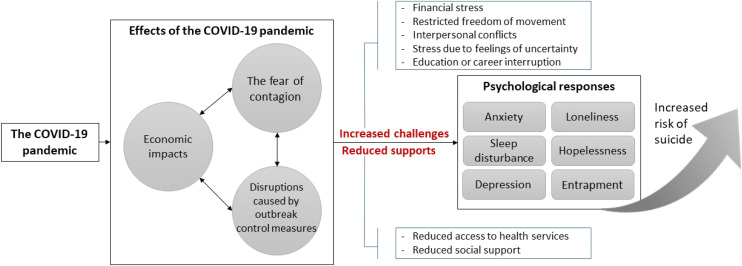
Possible processes linking the COVID-19 pandemic and suicide risk.

In total, 66.7% of the calls were made by female callers. Callers were aged between 19 and 75 years (Table 1). We identified three themes: (1) effects of the COVID-19 pandemic on society; (2) increased stress in the callers’ everyday lives, including increased challenges and reduced support; and (3) the callers’ psychological responses to stress that may contribute to increased suicide risk. Figure 1 illustrates the link between the three themes, showing the potential process underlying the pandemic’s impacts on suicide risk. In the following section, we first describe each theme and related factors and then use one caller’s experience to depict the links between the three themes. The details of the callers’ experiences have been altered to protect confidentiality.

### The Effects of the COVID-19 Pandemic on Society, Perceived by Callers as Three Rapid Environmental Changes

Based on callers’ reports, we identified three rapid environmental changes that represented the effects of the COVID-19 pandemic on society: the impacts on local economies, the fear of contagion, and disruptions caused by outbreak control measures.

#### Impacts on Local Economies

Nearly half of the callers (*n =* 26) reported that their income decreased due to mandatory unpaid leave or loss of job during the pandemic, as one caller said, “Now I only work two days per week. We have very few orders because the international cargo shipping is disrupted. We can’t import the materials from China, and we can’t export our products either” (ID#15). The affected industries included tourism, catering, transport services, and manufacturing industries. Casual laborers and sex workers also reported reduced income.

#### The Fear of Contagion

Around one-third of callers (*n =* 19) described concerns about the risk of infection for themselves and their family members. The callers described their actions in response to the fear of contagion, such as careful self-monitoring of any possible symptoms, implementing protective measures (e.g., measuring body temperature and increasing the frequency of handwashing and sanitizing), and avoiding leaving home or using public transport.

#### Disruptions Caused by Outbreak Control Measures

The disruptions that callers (*n =* 16) described included the consequences of border control, infection control measures (e.g., physical distancing and face masks), and home quarantine. For example, one caller indicated that the foreign domestic helper who was taking care of his family member with chronic diseases could not re-enter Taiwan due to strict boarder control measures (#26). Some callers reported that they or their significant others were unable to move freely between countries, as one caller said, “My boyfriend, who was an international student in Taiwan, went to China for the lunar new year holiday, but he couldn’t come back to Taiwan for months … I miss him very much, and it is truly devastating to me” (#43).

### The Pandemic’s Effects Led to Stress in Callers, Including Increased Challenges and Reduced Support

The effects of the pandemic described above led to increased challenges and reduced support experienced by callers in their everyday lives. These challenges included financial stress, restricted freedom of movement, interpersonal conflicts, stress due to feelings of uncertainty, and education or career interruption. Callers also reported reduced access to health services and reduced social support.

#### Financial Stress

Many callers’ income decreased as a result of unpaid leave or loss of job, while they still needed to pay their living expenses; this led to tremendous financial stress (*n =* 23). For example, one caller (a taxi driver) said, “I don’t have customers, but I still have to pay for the mortgage and pay for my kid’s food. I feel desperate” (#12). Although the Legislative Yuan (Taiwan’s legislative body) passed the Act on COVID-19 Prevention, Relief, and Recovery on February 25, 2020, to aid people whose livelihoods had been affected by the pandemic, some callers complained that they were unable to receive financial support as they did not meet the eligibility criteria. As one caller said, “I was told that I have to enroll in the employment insurance scheme to [be eligible to] apply for government cash subsidies” (#33).

#### Restricted Freedom of Movement

Some callers (*n =* 16) felt that their freedom of movement was substantially restricted due to the pandemic and outbreak control measures. One caller under the 14-day home quarantine mandated for all travelers returning to Taiwan said, “I feel bad in a confined space. I simply couldn’t go out” (#54). Although there was no lockdown in Taiwan, some facilities had infection control measures, which made the callers feel restricted. One caller who lived in a nursing home said, “We are not allowed to go out, and I felt I am under house arrest” (#59).

#### Interpersonal Conflicts

Some callers (*n =* 13) reported experiencing increased interpersonal conflicts, mostly with their family members. They reported having to spend much more time with their family members than previously after they moved back to Taiwan because of the pandemic, or because they had their working hours cut, or worse, they lost their jobs during the pandemic. Increased intensity of interactions with family members increased the frequency of quarrels. For example, one caller said, “Because of the pandemic, I hope my son could stay at home as much as possible [to avoid contracting the virus]. I argue with my son all the time, asking him not to go out; I am almost to have a breakdown” (#35). Some callers described how they felt suppressed while interacting with their family members during the pandemic, as two callers said, “I feel living at home is a painful experience because I have to hear hurtful words from them [family members] … I even have to lower my voice while crying because my father gets angry whenever he hears people crying” (#20); “I can’t tell them [family members] the truth about my financial stress. I don’t want to make them worried about me. However, I feel I am all alone in this struggle, and I can’t handle it anymore” (#27).

#### Stress Due to Feelings of Uncertainty

A few callers (*n =* 6) reported that they were stressed as they did not know how to plan for the future when the development of the pandemic remained unclear. One caller who originally ran her business in China and moved back to Taiwan temporarily due to the pandemic called the hotline because she felt extremely stressed about not knowing how she could move forward in life: “I feel so lost for the future … I don’t know what to do because I can’t see an end in sight” (#27).

#### Education or Career Interruption

A few callers (*n =* 3) described how their education and career plans were interrupted because of the pandemic. For example, one caller indicated that she planned to study in Japan from mid-2020 but was unable to go because of the travel ban implemented by the Japanese government: “I have worked so hard to start a new life, which I have always dreamed for, but now the opportunity is gone all of a sudden” (#24).

#### Reduced Access to Health Services

Some callers (*n =* 13) indicated that they could not access health services during the pandemic. Several callers complained that, as non-COVID-19-related patients, they felt that their medical needs were ignored because the government and hospitals reallocated resources to COVID-19-related care. Other callers said that they were hesitant to go see doctors or be hospitalized despite their medical needs. As one caller said, “I’m very nervous while thinking about going to the hospital because I think it would be crowded [and put me at risk of infection]. Last month, I was scheduled to have an ultrasound exam, and I chose not to go” (#52). Other callers with pre-existing mental illness reported that they were running out of medications and needed to renew their prescriptions, but they were hesitant to go to their regular medical appointments during the pandemic.

#### Reduced Social Support

Opportunities for social contact (e.g., community activities or family gatherings) were reported to be substantially reduced during the pandemic (*n =* 6). For example, one caller was asked by her son not to attend community events for elders because the son was worried about the risk of infection (#36).

### Psychological Responses to Stress That May Further Contribute to Increased Suicide Risk

We identified six categories of psychological responses to stress experienced by callers: anxiety, sleep disturbance, depression, loneliness, hopelessness, and entrapment. These psychological responses may contribute to increased suicide risk.

#### Anxiety

Feelings of anxiety, uneasiness, or nervousness were among the most common psychological responses identified in the data (*n =* 19). The callers felt anxious about the risk of infection. For example, one caller talked at length about the fear and worry of being infected, which led to multiple visits to clinics and hospitals within a short period. The anxiety further led to an increased frequency of self-harm behaviors. As one caller said, “I tried to distract myself from emotional pain by cutting myself … All of these struggles made me feel that I couldn’t control myself anymore, and thus I want to hurt myself” (#29).

#### Sleep Disturbance

Some callers (*n =* 18) complained about difficulties in falling asleep or maintaining sleep since they were worried about the pandemic and its effect, such as financial stress. The pandemic exacerbated sleep problems, particularly among those who previously experienced sleep disturbance. As one caller said, “I used to take only one sleeping pill before going to bed, but now I need a lot more” (#15).

#### Depression

Some callers (*n =* 14) indicated that they were experiencing low moods and aversion to activity, which increased their suicidal ideation. As one caller said, “Since the beginning of the pandemic, I’ve been feeling a fever and cold sweats all the time. I couldn’t eat and sleep, and I’m unable to laugh or smile or even cry. I feel that living has no meaning” (#53).

#### Loneliness

Some callers (*n =* 12) reported stress from having no company. For example, one caller felt much lonelier than before the pandemic as she had to take unpaid leave and could not meet colleagues as usual (#58). Some callers who were quarantined at home and had pre-existing mental health conditions reported feeling lonely, and this increased their suicidal ideation.

#### Hopelessness

Some callers (*n =* 11) reported no expectation of good or success, which increased their suicidal ideation. In around half of these callers, the main contributor to the feeling of hopelessness was financial stress. Several callers indicated that finding a job had never been easy for them, and it became nearly impossible during the pandemic. As a result, some reported feeling no motivation to live anymore. For example, one caller said, “I’d asked around, and almost all businesses were impacted by the pandemic. Their employees were also forced to take unpaid leave … It is just hard” (#8).

#### Entrapment

A few callers (*n =* 4) indicated that they wanted to escape from such a stressful situation, but the flight was blocked. For example, one caller said, “I don’t know if I should go back to China [to work]. If I go, my financial stress might be relieved, but if I get infected, the gain is not worth the loss. I feel I am stuck here” (#27).

These psychological responses to stress were particularly critical to some high-risk groups, such as individuals with pre-existing mental health conditions, those who had made previous suicide attempts, and those with precarious jobs. Below, we report a caller’s experiences to illustrate how the effects of the COVID-19 pandemic on society led to increased stress and psychological responses that, in turn, contributed to increased suicide risk. This caller indicated that his working hours were cut recently due to the pandemic (i.e., impacts on local economies), and he had been unable to pay his rent for several months (i.e., financial stress). He had been taking medications for his anxiety disorder, and one of his family members was seriously ill and might die soon. Such mounting pressure made him feel hopeless (i.e., psychological responses to stress) and think of suicide. He said:

I received the call [regarding his family member] from the hospital this morning. I really don’t know what to do. I don’t have money to pay [the bill], and it’s impossible to find other jobs now because all businesses are impacted [by the pandemic] … I feel I’m falling to pieces. I keep thinking about possible ways to kill myself. (#8)

## Discussion

Suicidal callers to Taiwan’s national suicide prevention hotline reported a range of ways the pandemic affected their mental health and suicide risk. The main effects of the pandemic on society were perceived as three rapid environmental changes: the impacts on local economies, the fear of contagion, and disruptions caused by outbreak control measures. As a result, the callers experienced stress from increased challenges, such as financial burden, restricted freedom of movement, interpersonal conflicts, feelings of uncertainty, and education or career interruption, as well as reduced access to health services and social support. In response, the callers presented with increased anxiety, sleep disturbance, depression, loneliness, hopelessness, and entrapment; these responses contributed to increased suicidal ideation in some callers. These findings inform possible mechanisms linking COVID-19-related factors to suicide risk.

The main effects of the COVID-19 pandemic that we identified in the conversations (i.e., impacts on local economies, the fear of contagion, and disruptions caused by outbreak control measures) are in keeping with findings from other studies of mental health effects of the pandemic and previous infectious diseases epidemics. We found that the COVID-19 pandemic’s economic impacts, even during the early months of the outbreak, could lead to increased suicide risk in affected individuals, as suggested by some researchers (Devitt, 2020; Gunnell et al., 2020; Niederkrotenthaler et al., 2020; Reger et al., 2020). Previous studies also consistently showed an increase in suicide rates following economic crises (Chang et al., 2013; Stuckler et al., 2009). In keeping with our findings, the fear of contagion was found to be a risk factor for suicide in Hong Kong during the 2003 SARS epidemic (Yip et al., 2010). For the current COVID-19 pandemic, Joshi et al. (2020) interviewed lifeline counselors to investigate callers’ responses to the pandemic in India and found the fear of contagion to be one of the callers’ main concerns. The fear of contagion was also found to have adverse influences on mental health and self-harm behaviors in the United Kingdom (Gillard et al., 2021; Hawton et al., 2021). Furthermore, two studies from the United Kingdom showed that outbreak control measures appeared to have negative influences on mental health in the general public (Pierce et al., 2021) and people with pre-existing mental health conditions (Gillard et al., 2021).

We identified a range of stressors experienced by hotline callers during the COVID-19 pandemic, in keeping with findings from other studies (i.e., financial burden, restricted freedom of movement, interpersonal conflicts, feelings of uncertainty, education/career interruption, and reduced access to health services and social support). Financial stress was found to be among the most frequently reported challenges associated with suicidal ideation and behavior during the pandemic (Elbogen et al., 2021; Gratz et al., 2020; Hawton et al., 2021). Similar to our findings, other studies reported the following risk factors for suicidal ideation and behavior during the pandemic: restricted freedom of movement (Dsouza et al., 2020; Gillard et al., 2021; Hawton et al., 2021), interpersonal conflicts (Gillard et al., 2021; Hawton et al., 2021; Joshi et al., 2020; Zhao et al., 2021), feelings of uncertainty (Brenner & Bhugra, 2020), education or career interruption (Gillard et al., 2021; Hawton et al., 2021), reduced access to health services (Gillard et al., 2021; Hawton et al., 2021; Joshi et al., 2020; Zhou et al., 2020), and reduced social support (Gillard et al., 2021; Hawton et al., 2021; Leaune et al., 2020; Na et al., 2021). In keeping with previous studies, some of the risk factors, such as conflicts with family members, appeared to be associated with strained family connections in Taiwanese families (Tzeng et al., 2010).

We identified six categories of psychological responses that may contribute to suicidal ideation among hotline callers. In the context of the COVID-19 pandemic, other studies also showed possible relationships between these psychological responses and increased suicide risk – they reported an association of suicide risk with anxiety (Brenner & Bhugra, 2020; Czeisler et al., 2020; Hawton et al., 2021), sleep disturbance (Hawton et al., 2021), depression (Czeisler et al., 2020; Hawton et al., 2021), loneliness (Antonelli-Salgado et al., 2021; Dsouza et al., 2020; Elbogen et al., 2021; Hawton et al., 2021; Na et al., 2021), hopelessness (Brenner & Bhugra, 2020), and entrapment (Brenner & Bhugra, 2020; Hawton et al., 2021). These psychological responses are in line with the components of psychological models of suicidal behavior (O'Connor & Nock, 2014), which propose that the emergence of suicidal ideation could be driven by feelings of entrapment (O'Connor & Kirtley, 2018), isolation and loneliness (Joiner, 2007; Van Orden et al., 2010), hopelessness (Beck et al., 1985; Joiner Jr. & Rudd, 1996), and mental conditions such as anxiety and depression (Mann, 2003; Mann et al., 1999).

We noticed that several factors highlighted by Gunnell et al. (2020) and Niederkrotenthaler et al. (2020) were less salient in our findings, including bereavement due to COVID-19, domestic violence, alcohol consumption, irresponsible media reporting, and changes in access to means of suicide. Over the study period, there were only seven COVID-19 deaths in Taiwan; this number was much smaller than that in many other countries then, and this would limit the impact of bereavement. Domestic violence and alcohol consumption, which may increase during lockdowns, were shown to influence self-harm risk in a study from the United Kingdom (Hawton et al., 2021), while Taiwan did not implement lockdowns during the study period. In the callers’ conversations, some callers mentioned the methods for suicide that they considered (e.g., jumping from high buildings or overdose), while no further information about any changes in access to these means was provided. No callers reported that media reporting of suicide had influenced their suicidal ideation. Further research is needed to investigate the effect of these factors on suicide risk across settings with various levels of pandemic severity and lockdowns.

Compared to previous studies, our study provided more information regarding the impacts of border control and sleep disturbance. Several studies focused on the effects of outbreak mitigation measures such as lockdown and quarantine (Dsouza et al., 2020; Gillard et al., 2021; Pierce et al., 2021; Zhou et al., 2020), while our data indicated that strict border control might also markedly influence some people’s daily lives and their mental health. Compared to many other countries, Taiwan’s government implemented relatively tight border control measures in response to the pandemic; for example, most noncitizens were barred from entering the country, and all travelers entering Taiwan were required to home quarantine for 14 days. Furthermore, we noticed that sleep disturbance was frequently reported by callers (18 of 60), while it was not so commonly highlighted in previous studies. Lin et al. (2020) analyzed Google Trends data and showed an association between the number of COVID-19 deaths and the increase in online search using the term “insomnia” across 19 countries during the early months of the pandemic. Further research is needed to investigate the prevalence of sleep disturbance during the pandemic and how it may contribute to the occurrence of suicidal ideation.

The findings from this study have implications for practice, policy, and research. The themes that we identified revealed the possible process linking the effects of the COVID-19 pandemic to stress encountered by vulnerable individuals, their psychological responses, and in turn, increased suicide risk. These provide hotline helpers and clinicians a reference when assessing risk and providing support and treatments for vulnerable individuals in the context of the pandemic. In addition to applying the skill sets that hotline helpers are already familiar with, such as active listening (Woodward & Wyllie, 2016), helpers could also support the callers based on a better understanding of possible psychological responses to the pandemic’s impacts and referring them to appropriate resources that can help alleviate these impacts. For suicide prevention and mental health protection strategies during the pandemic, suicide prevention hotlines could not only be an important source of emotional support but also serve as a useful source of practical information such as government resources (e.g., financial assistance) to relieve stressors that would impact mental health and increase suicide risk. It is crucial for governments and private sector leaders to provide sufficient support to ensure the maintenance of suicide prevention hotline services during such public health emergencies. Our findings could also inform further studies aimed at identifying mechanisms underlying the pandemic’s impacts on suicide risk at different stages of the pandemic (Holmes et al., 2020; Niederkrotenthaler et al., 2020).

### Strengths and Limitations

The key strength of this study is that, through qualitative analysis, we were able to reveal the nuance and complexity of the pandemic’s impacts on mental health and suicide risk by exploring how different impacts, consequences, and individuals’ responses could interact to influence suicide risk. Another strength is that we were able to explore the concerns and lived experiences of hotline callers through analyzing their conversations with helpers; by contrast, the opportunities to conduct face-to-face interviews with vulnerable individuals became limited during the outbreak.

There are several limitations of this study. First, we were only able to explore the experiences of those who sought help by calling the hotline. Furthermore, our analysis was restricted to calls that hotline helpers had indicated as related to the COVID-19 pandemic. Second, the 60 eligible calls included in our analysis were from a small subsample of all individuals who called the hotline during the study period. Moreover, we were unable to know if these calls were made by 60 different individuals as some calls could be from the same persons. Third, due to the nature of the hotline, the helpers would not lead the conversation following any interview guides for research purposes. We thus could only analyze recorded free-flowing conversations between the helpers and callers. We were unable to explore issues not discussed in the conversations. Fourth, we only included and analyzed calls from the first fourth months of the pandemic; however, the COVID-19 outbreak in Taiwan in 2020 was mostly limited to these months. Last, the scale of the outbreak in Taiwan was relatively small compared to those in many other countries, and we were unable to explore other impacts (e.g., bereavement and domestic violence) that were more salient in countries more severely affected by the pandemic. Future research is needed to conduct in-depth interviews with affected individuals regarding their perceived impacts at different stages of the COVID-19 pandemic concerning suicide risk.

### Conclusions

There are multiple complex processes through which the COVID-19 pandemic may impact mental health and suicide risk. The findings from this study could support suicide prevention responses during the pandemic by informing hotline helpers’ and clinicians’ strategies to assess and support suicidal individuals. The relationships between the three themes that we identified in this study could also inform further research to investigate the mechanisms underlying the impacts of the pandemic on mental health and suicide risk.

## Notes

**Publication Ethics:** Ethical approval for this study was granted by The Research Ethics Committee C, National Taiwan University Hospital (Reference No. 202004065RINC). As part of the hotline practice, the helpers asked for and obtained the callers’ consent for recording. The ethics committee deemed our study as secondary data analysis and agreed that our data management and analysis could ensure the protection of anonymity and confidentiality.
